# Human Body–Electrode Interfaces for Wide-Frequency Sensing and Communication: A Review

**DOI:** 10.3390/nano11082152

**Published:** 2021-08-23

**Authors:** Kurian Polachan, Baibhab Chatterjee, Scott Weigand, Shreyas Sen

**Affiliations:** 1 School of Electrical and Computer Engineering, Purdue University, West Lafayette, IN 47906, USA; bchatte@purdue.edu (B.C.); shreyas@purdue.edu (S.S.); 2 Eli Lilly and Company, Indianapolis, IN 46285, USA; weigand_scott@lilly.com

**Keywords:** body–electrode interface, biopotential sensing, human body communication

## Abstract

Several on-body sensing and communication applications use electrodes in contact with the human body. Body–electrode interfaces in these cases act as a transducer, converting ionic current in the body to electronic current in the sensing and communication circuits and vice versa. An ideal body–electrode interface should have the characteristics of an electrical short, i.e., the transfer of ionic currents and electronic currents across the interface should happen without any hindrance. However, practical body–electrode interfaces often have definite impedances and potentials that hinder the free flow of currents, affecting the application’s performance. Minimizing the impact of body–electrode interfaces on the application’s performance requires one to understand the physics of such interfaces, how it distorts the signals passing through it, and how the interface-induced signal degradations affect the applications. Our work deals with reviewing these elements in the context of biopotential sensing and human body communication.

## 1. Introduction

Both biopotential sensing and human body communication (HBC) use electrodes in contact with the body. Biopotential sensing uses these electrodes to acquire signals from the body [1,2]. Human body communication [3,4,5,6,7,8,9,10,11,12,13], on the other hand, uses them to transfer signals from one part of the body to the other. Unlike radiative communication that uses airwaves, HBC, specifically electro-quasistatic HBC (EQS-HBC), couple signals and confine them within the human body [14]. Once coupled, the signal is present anywhere on the body surface and can be received from any location on the body. This leads to similarities between biopotential sensing and HBC, albeit at different frequencies, in terms of how they use the body–electrode interface. This review highlights the underlining principles of body–electrode interfaces with similarities and differences for sensing and communication.

**Figure 1:**
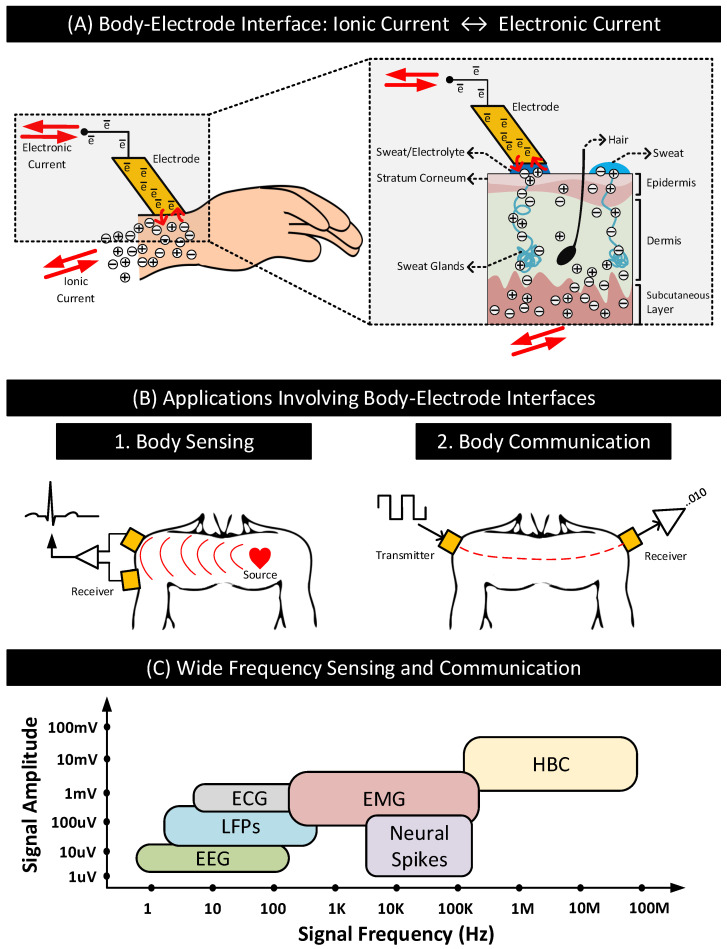
(**A**) A typical body–electrode interface: ionic current in the body is converted to electronic current and vice versa at the interface. (**B**) Differential sensing of signals from the body and communication of data signals through the body using electrodes. (**C**) Approximate signal amplitudes and frequency ranges of biopotential and HBC signals.

**Figure 2:**
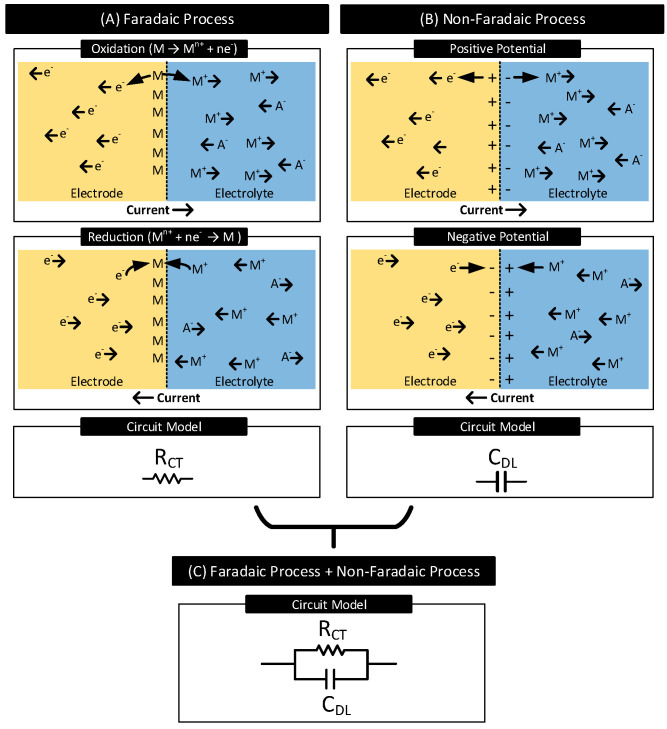
(**A**) Faradaic Process: Oxidation and reduction reactions occur at the electrode–electrolyte interface involving atoms in the electrode (*M*) and cations ($M^{+}$) in the electrolyte, resulting in charge transfer across the interface. Oxidation dominates when current flows from electrode to electrolyte, and reduction dominates when the current direction is the opposite. Resistor $R_{CT}$ models the faradaic process of current transfer. (**B**) Non-Faradaic Process: Charges do not cross the interface; instead, they accumulate across the interface. When the current flows from electrode to electrolyte, the electrode gains a positive charge near the interface and the electrolyte a negative charge. Charges reverse when the direction of the current is the opposite. Capacitor $C_{DL}$ models the non-faradaic process of current transfer. (**C**) Resistor $R_{CT}$ in parallel to capacitor $C_{DL}$ models the interfaces which support both faradaic and non-faradic processes of current transfers.

Figure 1(B-1) shows the schematic of differential sensing of biopotential signals using a pair of electrodes, and Figure 1(B-2) shows the schematic of transferring data through the human body using an electrode acting as a transmitter and another as a receiver. In both cases, the electrodes in contact with the body create an interface called the body–electrode interface that serves a specific purpose. The interface facilitates current flow between the body and electrode/electronic circuits, enabling signal measurements, communication, or both. The interface is essential because the body carries current mainly through ions present in bodily fluids, while electrons carry the current in the electronic circuit. The interface, therefore, functions as a transducer, converting ionic current in the body to electronic current in the circuits and vice versa [1,2]. Figure 1A depicts this behavior. The figure shows an electrode residing on the surface of human skin. When current flows from the body to the electrode, ions from the inner bodily fluids traverse through different layers of the skin to reach the surface. These ions contact the electrode through an electrolyte layer (e.g., electrolyte gel or body sweat). The conversion of the ions to electrons happens at the contact, resulting in the flow of electrons in the electrode, wire, and connected electronic circuit. The process reverses when current flows from the electrode to the body.

An ideal body–electrode interface should have the characteristics of an electrical short, i.e., the transfer of ionic currents and electronic currents across the interface should happen without any hindrance. However, in practice, that is not the case. Practical body–electrode interfaces often have definite impedances and potentials that hinder the free flow of currents. In sensing and communication applications, such interfaces can degrade the quality of the signals passing through the interface, affecting the application’s performance. The review of the body–electrode interface and its impact on sensing and communication is of importance today due to emerging applications such as on-body health monitors that combine biopotential sensing with human body communications. On-body health monitors may use HBC [3,8,15] as a replacement to radiative technologies such as Bluetooth to transmit biopotential signals representing the subject’s health conditions to an aggregator [16,17,18,19,20,21]. Health monitors enabled with HBC transmit data to the aggregator when the latter is in direct contact with the body, which is always the case when the aggregator is also an on-body device such as a smartwatch. In comparison to radiative technologies, HBC consumes significantly lower power extending the battery life of these devices and provides improved physical security to the sensitive health data by confining the signals within the subject’s body [8,14,22]. Further, HBC can operate at frequencies way above the signal frequencies of most common biopotential signals listed in Table 1, eliminating the possibility of inter-signal (sensing and communication) interferences. Figure 1C shows the signal frequency ranges of the biopotential signals in Table 1 and how they compare to HBC [3,23,24]. The biopotential signal frequencies are often restricted to ≤ 100 KHz, while HBC can work well above 100 KHz up to 100 MHz. The wide-frequency operating range of HBC is furthermore appealing to health monitors. It allows the devices to tune the HBC operating frequency to optimize power consumption, data rate, or both.

Rectifying or minimizing the impact of practical body–electrode interfaces on the applications of the above kind requires one to understand the details of the following elements—the physics of the body–electrode interface, how the interface distorts the signals passing through it, and how the interface-induced signal degradations affect the sensing of biopotential signals and HBC. To the best of our knowledge, we do not find a consolidated review of these elements in the literature. Through this work, we attempt to address this gap.

We organize this paper as follows: Section 2 describe the body–electrode interface. Specifically, the physics of the interface and how the interface distorts signals passing through it. Section 3 and Section 4 describes biopotential sensing and human body communication, respectively, and discuss how the signal distortions created by the body–electrode interface impact the quality and performance of the sensing and communication. Section 5 summarizes our contributions and concludes this paper.

## 2. Body–Electrode Interface

This section first discusses the physics of the body–electrode interfaces for two different cases: (i) implanted electrodes that are in direct contact with body fluids and (ii) surface electrodes that reside on the skin’s surface. The interface characteristics of the implanted electrode are similar to that of the classic electrode–electrolyte interface. Thus, in the first part of this section, we discuss the electrode–electrolyte interface and its properties. The interface characteristics of the surface electrode also depend on the skin properties and electrode configuration, which we discuss in the second part of this section. This section further discusses how the body–electrode interface distorts signals and describes the form factors and materials of some common surface and implanted electrodes.

### 2.1. Electrode–Electrolyte Interface

The conversion of ionic current to electronic current and vice versa across the interface happens via two types of current transfer mechanisms: faradaic and non-faradaic. The first part of this section discusses these current transfer mechanisms. The second part of this section introduces half-cell potential, which is the difference in potential between the electrode and electrolyte when they contact each other. The third part of this section discusses the classification of electrodes based on their current transfer characteristics and the equivalent circuit models for their interfaces.

#### 2.1.1. Current Transfer Mechanisms

For an electrode that is in direct contact with the bodily fluids (e.g., implanted electrodes), current transfer mechanisms across the electrode–body interface are similar to those in an electrode–electrolyte interface. The similarity exists because the bodily fluids act similar to electrolytes rich in positively charged cations and negatively charged anions.

For an electrode in contact with an electrolyte, the current is carried across the interface by two main processes: faradaic process and non-faradaic process [29].

1. Faradaic Process: In this process of current transfer, charges (electrons/ions) cross the electrode–electrolyte interface by electrochemical reactions: oxidation and reduction [1,2].
  Oxidation occurs when atoms in the electrode (*M*) leave electrons behind and goes into the electrolyte as a cation ($M^{+}$) or when anions ($A^{-}$) in the electrolyte transform to a neutral atom, leaving electrons to the electrode. The following reactions represent oxidation:
  $$M\overset{}{\to }M^{n+}+ne^{-}$$
  $$A^{m-}\overset{}{\to }A+me^{-}$$
  where *n* is the valence of *M* and *m* is the valence of *A*. Reversal of oxidation reactions results in reduction reactions. The following reactions represent reduction:
  $$M^{n+}+ne^{-}\overset{}{\to }M$$
  $$A+me^{-}\overset{}{\to }A^{m-}$$
  Figure 2A shows the oxidation and the reduction reactions involving *M* and $M^{+}$ and the direction of flow of charge carriers. In the presence of a driving current source, $e^{-}$ in the electrode moves in the opposite direction as the current. Charge carriers $A^{-}$ and $M^{+}$ in the electrolyte move in the opposite and same direction as the current, respectively. Oxidation reactions dominate when the current flows from electrode to electrolyte, and reduction reactions dominate when the current direction is the opposite.
  In a faradaic process, the charge transfer across the interface obeys Faraday’s law, i.e., the amount of chemical reactions resulting in charge transfer across the interface is proportional to the current that flows through the interface [30]. The electrode–electrolyte interface undergoing the faradaic process thus behaves like a resistor [29]. This resistor is known as the charge-transfer resistor, $R_{CT}$ [31,32].
2. Non-Faradaic Process: In this process of current transfer, charges never cross the interface. Rather, they accumulate across either side of the interface, polarizing the electrode. See Figure 2B. When current flows from electrode to electrolyte, near the interface, positive charges accumulate in the electrode ($e^{-}$ moves away from the interface), and negative charges accumulate in the electrolyte ($M^{-}$ moves away from the interface, $A^{-}$ moves closer to the interface) [33]. These charge redistributions result in the electrode acquiring a positive potential that increases with time. The charge and the voltage polarities reverse when the direction of the current is the opposite. The accumulation of charges across the interface results in the interface acting as a capacitor known as the interface double-layer capacitor, $C_{DL}$. The current transfer across the interface is due to the displacement current resulting from the charging and discharging of $C_{DL}$ [29].

Both faradaic and non-faradaic processes contribute to current transfer across many electrode–electrolyte interfaces. Figure 2C shows the circuit model for such interfaces. It consists of the charge-transfer resistor ($R_{CT}$) in parallel with the double-layer capacitor ($C_{DL}$).

#### 2.1.2. Half-Cell Potential

In many electrode–electrolyte interfaces, in the beginning, i.e., as soon as the electrode makes the first contact with the electrolyte, charges (ions/electrons) cross the interface through oxidation and reduction reactions; this happens even in the absence of any external driving current source. The rates of these reactions depend on the electrode characteristics, electrolyte concentration, and ambient conditions (e.g., temperature). Any imbalance in these oxidation and reduction reactions rates can cause unequal charge transfers across the interface, disturbing the neutrality of charges in the region and resulting in the electrode acquiring a different potential than that of the electrolyte, which favors the weaker reaction.

**Figure 3:**
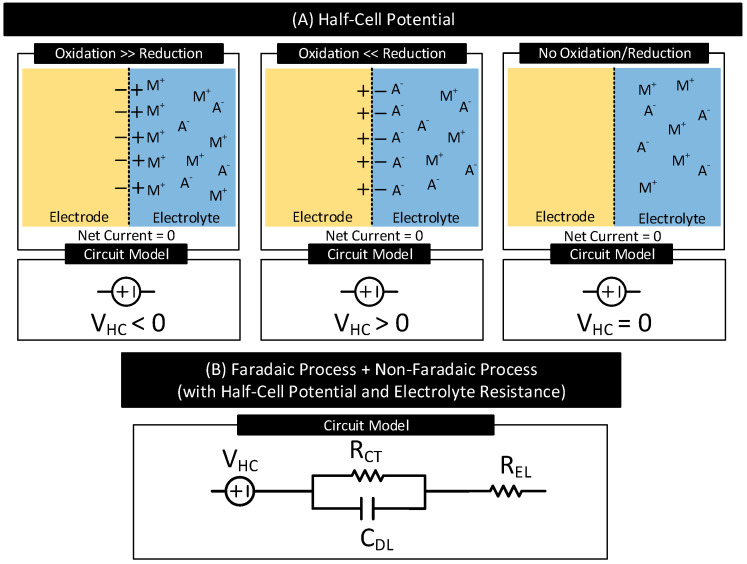
(**A**) Electrode half-cell potential (also known as contact potential), $V_{HC}$, for three different cases. $V_{HC}$ is positive if at the interfaces, in the beginning, oxidation reactions dominate reduction reactions. $V_{HC}$ is negative if at the interfaces, in the beginning, reduction reactions dominate oxidation reactions. $V_{HC}$ is zero in the absence of oxidation and reduction reactions. (**B**) Circuit model of an electrode–electrolyte interface supporting both faradaic and non-faradaic processes of current transfer with half-cell potential, $V_{HC}$, and electrolyte resistance, $R_{EL}$.

For instance, in interfaces where oxidation reactions dominate over reduction reactions, near the interface, positive charges ($M^{+}$) accumulate at the electrolyte, and negative charges ($e^{-}$) accumulate at the electrode, resulting in a difference in potential between the electrode and the electrolyte. See Figure 3. Here, an increase in the concentration of ($M^{+}$) in the electrolyte near the electrode reduces the rate of further oxidation reactions and increases the rate of further reduction reactions. The difference in potential increases until it drives the difference in the reaction rates to zero, which happens at equilibrium. The difference in electrode–electrolyte potential at equilibrium is known as the half-cell potential (or contact potential), $V_{HC}$ [2].

Figure 3A shows the polarity of $V_{HC}$ for three different electrode–electrolyte interfaces. $V_{HC}$ is (i) negative in interfaces if, in the beginning, oxidation reactions dominate reduction reactions, (ii) positive in interfaces if, in the beginning, reduction reactions dominate oxidation reactions, and (iii) zero in interfaces that do not support oxidation and reduction reactions. Table 2 lists $V_{HC}$ of several electrodes referenced to the standard hydrogen electrode under standard temperature (25 ${}^{\circ }$C), pressure (1 atm) and electrolyte concentration (1 mol dm^−3^) [1]. When the temperature, pressure, or electrolyte concentration changes from their standard values used for the measurements, $V_{HC}$ of the electrodes varies from their reported values in Table 2 in accordance with the Nernst equaiton [1].

Figure 3B shows the circuit model of an electrode–electrolyte interface undergoing both faradaic and non-faradaic process of current transfer and with half-cell potential, $V_{HC}$, and electrolyte resistance, $R_{EL}$. Figure 6A shows the circuit model of the electrode–body interface for an implanted electrode. The circuit model is similar to Figure 3B with $R_{EL}$ replaced with $R_{DSL}$ which represents the resistance of the inner body fluids.

#### 2.1.3. Electrode Polarization

In many electrode–electrolyte interfaces, when direct current passes through the interface, the potential of the electrode changes from its equilibrium potential of $V_{HC}$. The phenomenon is called electrode polarization, and the difference in potential is called overpotential [2]. The current transfer mechanisms at the electrode–electrolyte interface dictate the characteristics of the electrode polarization. Based on the current transfer mechanisms at the electrode–electrolyte interface and thus the resultant polarization characteristics, electrodes are grouped into the following types.

- Current Transfer via Faradaic Process
  - Non-Polarizable (or Charge Transfer) Electrodes: The current transfer across the interface is solely due to the faradaic process. They have a non-zero charge-transfer resistance (i.e., $R_{CT}\neq 0$). The degree of polarization is proportional to $R_{CT}$.
  - Ideal Non-Polarizable Electrodes: They are non-polarizable electrodes with zero charge transfer resistance (i.e., $R_{CT}=0$). Their interface behaves like an electrical short. They offer no resistance to direct current flow and thus do not polarize the electrode, i.e., the electrode potential does not change from its equilibrium value.
- Current Transfer via Non-Faradaic Process
  - Ideal Polarizable Electrodes: The current transfer across the interface is solely due to the non-faradaic process. Their interface behaves like a capacitor and thus blocks direct current. The direct current causes the magnitude of the electrode potential to increase over time without limits.
- Current Transfer via Faradaic and Non-Faradaic Processes
  - General Polarizable Electrodes: In these electrodes, both faradaic and non-faradaic processes contribute to the current transfer across the interface. The degree of polarization depends on the values of the charge transfer resistance and double-layer capacitance of the interface.

**Figure 4:**
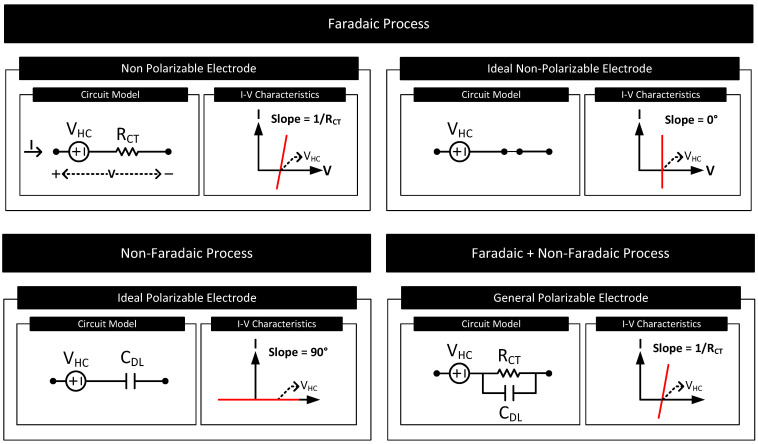
The circuit model and I-V characteristics of the electrode–electrolyte interface for electrodes of different polarization characteristics.

Figure 4 lists these electrode types, their interface circuit models and I-V characteristics. Although the I-V characteristics of the non-polarizable and general polarizable electrode look the same, in the latter case, after the application of direct current, it takes a definite amount of time decided by the product of $R_{CT}$ and $C_{DL}$ for the voltage across the interface to stabilize.

### 2.2. Electrode–Skin Interface

Often electrodes are not in direct contact with the body fluids as considered in Section 2.1. Instead, they reside on the surface of the skin as in Figure 1. Such electrodes are called surface electrodes. The characteristics of the electrode–body interface for the surface electrodes depend on the skin’s electrical characteristics and the electrode type. The first part of this section focuses on the electrical model of the skin, and the second part on the type of surface electrodes and the equivalent circuit models of their interfaces.

#### 2.2.1. Electrical Model of the Skin

**Figure 5:**
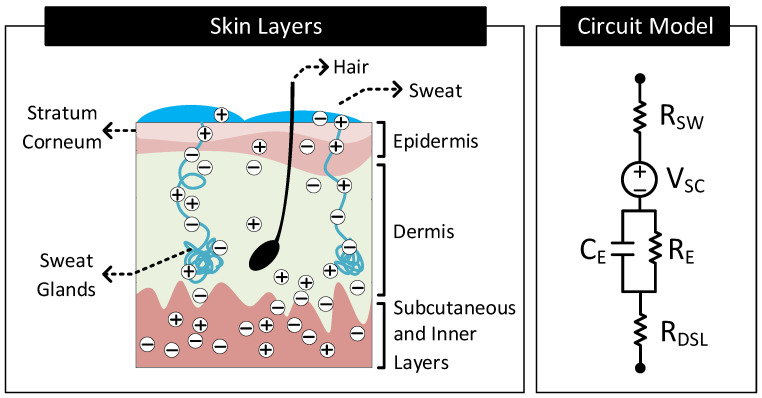
Different layers constituting the human skin and its equivalent circuit model.

Figure 5 shows different layers of the skin and its equivalent circuit model. The skin consists of three primary layers: epidermis, dermis, and subcutaneous layer. Inner layers dermis and subcutaneous are highly conductive and directly in contact with inner bodily fluids rich in ions. Resistor $R_{DSL}$ (≈$10k\Omega \;{\mathrm{cm}}^{-2}$ [34]) models these layers. The epidermis is the outermost layer of the skin. This layer consists of multiple sublayers, of which the outermost layer is called the stratum corneum. The stratum corneum is made mainly of dead cells and has the highest electrical resistance among the skin layers. The layer acts as a dielectric sandwiched between the conductive dermis layer and the conductive sweat layer on the skin surface. The capacitor $C_{E}$ models this structure.

Even though the stratum corneum has high electrical resistance, ions from inner bodily fluids still reach the skin surface and mix with the sweat. A part of this ion transport is through the pores in the stratum corneum. However, a significant portion of this ion transport happens through the sweat glands and hair foliages. The ions in the dermis layers diffuse into the sweat glands and hair foliages’ roots and move through the duct to the skin surface [35]. These conductive paths for the ions mimic a resistor, $R_{E}$, in parallel to $C_{E}$ [34]. The sweat on the surface of the skin is rich in ions and thus conductive. Resistor, $R_{SW}$, models this sweat layer.

Skin Potential: Similar to the potential difference ($V_{HC}$) that exists across an electrode–electrolyte interface, there can exist a potential difference across an electrolyte–electrolyte interface, known as liquid-junction potential [2]. The difference in potential exists across the electrolyte–electrolyte interface when the electrolytes have different ionic concentrations, and a semipermeable membrane separates them. Specific to Figure 5, the stratum corneum behaves like a semipermeable membrane separating the electrolytes, sweat, and body fluids of different ionic concentrations. This arrangement, therefore, results in a difference in potential to exist across the stratum corneum. This difference in potential is known as the skin potential. The voltage source, $V_{SC}$ (≈−30mV [36]), models this difference in potential.

#### 2.2.2. Types of Surface Electrodes

**Figure 6:**
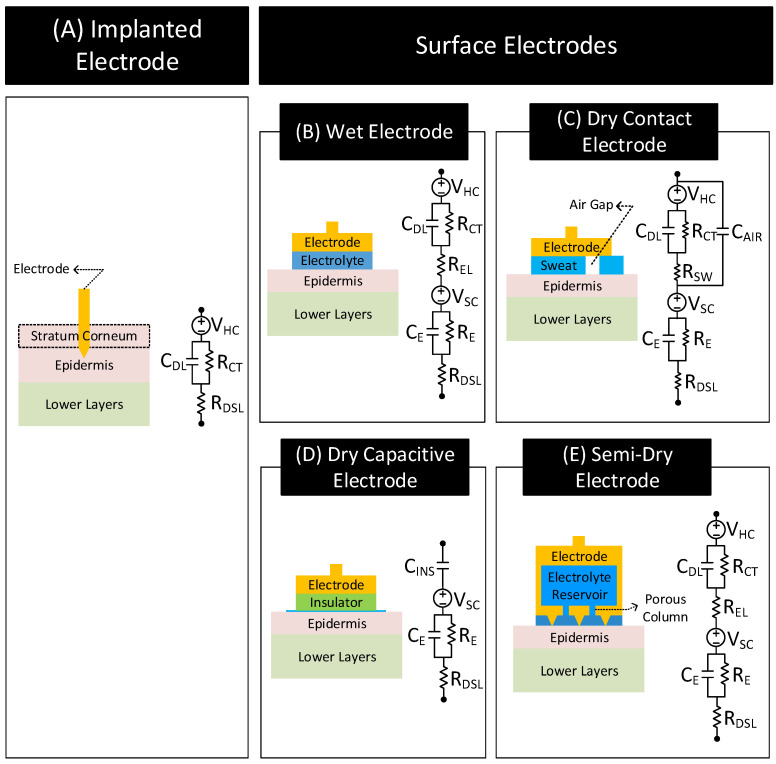
Layer stack and circuit model of the electrode–body interface for different electrode types. Inspired from [32,34,39,44,50,52,53,54,55,56,57,58].

Depending on how the electrode makes contact with the skin surface, surface electrodes are grouped into four types:

1. Wet Electrode: These electrodes make contact with the skin through an explicitly added electrolyte layer on the skin surface. The electrolyte layer hydrates the skin layer and improves the electrical conductivity of the stratum corneum [34]. Figure 6B shows the layer stack and circuit model of the electrode–skin interface for a wet electrode. Model parameters $V_{HC}$, $R_{CT}$ and $C_{DL}$ are the half-cell potential, charge transfer resistance, and double-layer capacitance, respectively, of the electrode–electrolyte interface. $R_{EL}$ is the resistance of the electrolyte layer. Model parameters $V_{SC}$, $R_{E}$ (≈10–100 KΩ cm${}^{-2}$ [34]), $C_{E}$ (≈10–50 nF cm${}^{-2}$ [34]) and $R_{DSL}$ constitute the skin potential and impedance components.
2. Dry-Contact Electrode: These electrodes make direct contact with the skin surface without relying on an externally applied electrolyte [34]. Here, the moisture or sweat on the skin surface plays the role of the electrolyte, hydrating the skin surface and lowerering the skin impedance [37,38]. However, since the moisture and sweat distribution is uneven, varies over time, and often has less ion concentration than off-the-shelf electrolytes, the electrode to skin impedance with dry electrodes is higher than the wet electrode’s case and changes over time. Additionally, the electrode–skin interface also traps air bubbles, blocking parts of the electrode surface from contacting the skin surface directly, adding a capacitive component to the interface impedance. The interface impedance also depends on the pressure applied to the electrode [39,40,41]. The dependency of the interface impedance on sweat, trapped air bubbles, and pressure mean that the electrode–skin characteristics of these electrodes vary with time, ambient conditions (e.g., temperature, humidity), subject’s movement, and electrode form factor (e.g., rigid or flexible electrodes) [42,43]. Figure 6C shows the layer stack and circuit model of the electrode–skin interface for a dry contact electrode. Model parameters $V_{HC}$, $R_{CT}$, and $C_{DL}$ are the half-cell potential, charge transfer resistance, and double-layer capacitance, respectively, of the electrode–sweat interface. $R_{SW}$ is the resistance of the sweat layer. $C_{AIR}$ models the capacitance of the electrode that does not make contact with the skin due to trapped air bubbles. Model parameters $V_{SC}$, $R_{E}$ (≈30–1000 KΩ cm${}^{-2}$ [34]), $C_{E}$ (≈10–50 nF cm${}^{-2}$ [34]) and $R_{DSL}$ constitute the skin potential and impedance components.
3. Dry-Capacitive Electrode: These electrodes do not make direct contact with the skin [34,44]. They are electrically isolated from the skin through an insulating layer (e.g., air or clothes). These electrodes eliminate the chance of skin irritation, protect the body from any electrical mishaps, and are easy to clean [45]. However, they are prone to motion artifacts, i.e., their contact impedances with the skin vary with the subject’s movements [44]. Figure 6D shows the layer stack and circuit model of the electrode–skin interface for these electrodes. The parameter $C_{INS}$ (≈1 pF–10 nF cm${}^{-2}$ [34]) models the insulation layer between the electrode and the skin surface. Since there does not exist any electrode–electrolyte interface, the circuit model of these electrodes does not include $V_{HC}$, $R_{CT}$, $C_{DL}$ or $R_{EL}$ typical of an electrode–electrolyte interface. Model parameters $V_{SC}$, $R_{E}$ (≈100–1000 KΩ cm${}^{-2}$ [34]), $C_{E}$ (≈10–50nF cm${}^{-2}$ [34]) and $R_{DSL}$ constitute the skin potential and impedance components.
4. Semi-Dry Electrode: These electrodes are placed directly on the skin surface like a dry electrode without direct application of electrolyte. However, unlike a dry electrode, a semi-dry electrode has inbuilt reservoirs which store electrolytes. While in contact with the body, the semi-dry electrode slowly releases the electrolyte in its reservoir to the skin surface (e.g., through porous columns in the reservoir) [46,47,48,49,50,51]. The released electrolyte hydrates the skin and improves the conductivity of the stratum corneum similarly to a wet electrode. Figure 6E shows the layer stack and circuit model of a semi-dry electrode that releases electrolyte in its reservoir through porous columns. The circuit model is similar to the wet electrode with the following difference. $R_{EL}$ for the semi-dry electrode is generally higher due to higher electrolyte resistance at the porous columns [50].

Apart from the electrode type, the properties of the electrode–skin interface also depend on the amount of electrolyte present, the contact pressure, and the skin preparations as follows.

- Electrolyte Amount: The electrolyte at the electrode–skin interface hydrates the stratum corneum layer of the skin and improves the contact impedance. Improvement in contact impedance with electrolytes is evident when comparing the contact impedance of wet/semi-dry electrodes with dry electrodes. The contact impedance of the dry electrode is approximately 50-fold higher than the wet/semi-dry electrodes that use electrolytes [59]. Using higher amounts of electrolytes is also desirable as it reduces the contact impedance by lowering both $R_{EL}$ and $R_{E}$. In particular, higher amounts of electrolytes can easily penetrate the skin layers and hydrate the layers lowering $R_{E}$ [50]. However, having higher amounts of electrolytes can cause discomforts to the user and cause possible inter-electrode shorts in cases where the electrodes are close to each other.
- Skin Preparation: Often, to improve the electrode–skin contact, skin preparations are performed at the electrode sites before placing the electrodes [60,61]. Skin preparations commonly involve cleaning, shaving, and abrasion. Cleaning and shaving help remove dirt and hairs from the skin, while abrasion removes the topmost stratum corneum layer. Skin preparation helps in reducing the interface impedance. For instance, performing skin abrasion can reduce interface impedance in dry-contact electrodes by about 80% [59]. Nevertheless, skin preparations can be time-consuming, costly, and painful to the subjects involved.
- Contact Pressure: Electrode–skin contact also improves with the application of pressure on the electrodes. The effect is significant, specifically in dry and semi-dry electrodes, where pressure significantly reduces the contact impedance [39,40,41,50]. Unlike wet electrodes, dry and semi-dry electrodes do not maintain stable contact with the skin due to the absence or relatively low amounts of electrolytes. For instance, authors in [50] find a decrease in contact impedance of 71% and 35% in dry-contact and semi-dry electrodes, respectively, with mild pressure. Application of pressure in these electrodes lowers both $R_{EL}$ and $R_{E}$. The decrease in $R_{EL}$ is due to close contact of the electrode with the skin. In contrast, the drop in $R_{E}$ is due to compression of skin layers shortening the ionic-current channel and due to porous columns of the semi-dry electrode penetrating the top layers of the skin [50].

Note: In the case of the implanted electrode, the tip of the electrode penetrates the stratum corneum layer of the epidermis and comes in direct contact with body tissues and bodily fluids. Direct contact of body tissues allows them to transfer signals with less loss of frequency content in comparison to surface electrodes [1]. Figure 6A shows the layer stack and circuit model for the implanted electrode. The model consists of $V_{HC}$, $R_{CT}$, and $C_{HL}$, the electrode–electrolyte (electrolyte ≡ body fluids) half-cell potential, the charge-transfer resistance, and the double-layer capacitance, respectively. In the model, the electrolyte resistance $R_{EL}$) is replaced with $R_{DSL}$ since the body fluids act as the electrolyte. Since the electrode bypasses the stratum corneum layer and contacts body fluids directly, the circuit model of these electrodes does not include skin potential and impedance components: $V_{SC}$, $R_{E}$, $C_{E}$ or $R_{SW}$.

Note: Invasive electrode–brain electrode falls under the category of implanted electrode. Its interface layer stack and circuit model are similar to the layer stack and circuit model of the implanted electrode shown in Figure 6A [62].

### 2.3. Signal Distortions: Types and Causes

The body–electrode interface can distort signals passing through the interface. The signal distortions can often be a cause of concern in sensing and communication applications. It is thus essential to understand the factors that can contribute to signal distortion. This section discusses the types of signal distortion and their causes.

**Figure 7:**
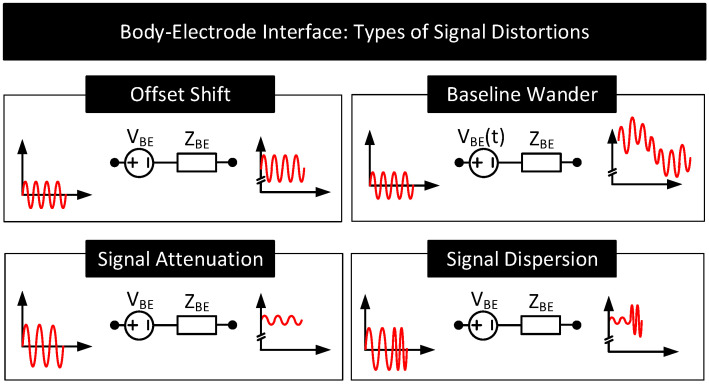
Types of distortions experienced by a signal passing through the body–electrode interface. A voltage source, $V_{BE}$, in series with the impedance, $Z_{BE}$, models the interface.

Figure 7 shows the possible types of distortions a signal experiences when traveling through the body–electrode interface. In the figure, the voltage source $V_{BE}$ and impedance $Z_{BE}$ in series model the contact potential and impedance of the interface, respectively.

**Figure 8:**
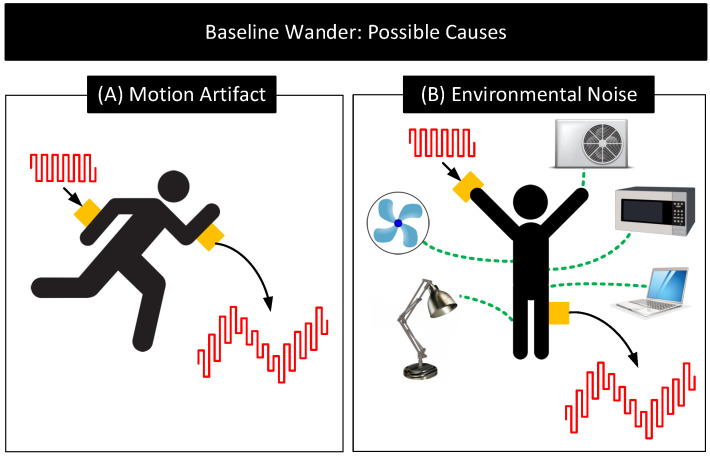
Potential causes for baseline wander. (**A**) Motion Artifact: Distortion of signals due to subject’s movement (**B**) Environmental Noise: Distortion of signals due to the coupling of signals to the body and electrodes from environmental noise sources [71].

1. Offset Shift: For the signals passing through the interface, the electrode half-cell potential ($V_{HC}$) together with skin potential ($V_{SC}$) behaves like a series DC voltage source represented in Figure 7 as $V_{BE}$. The presence of $V_{BE}$ shifts the DC offset of the passing signals. Since $V_{HC}$ is in hundreds of millivolts, often the offset shift is many folds higher than the sensing and communication signal amplitudes which are approximately a few microvolts to millivolts in range. The amount of offset shift depends on the electrode and its position on the body. The dependency is because $V_{HC}$ vary with the electrode (see Table 2) and $V_{SC}$ vary with the location on the body [36].
2. Baseline Wander: The offset $V_{BE}$ added to the signals is often not a constant; it varies slowly with time (i.e., $V_{BE}$→$V_{BE}\left(t\right)$) resulting in the baseline of the signal shifting over time. This phenomenon is called baseline wander. Following are the factors that can potentially cause baseline wander.
  (a) Motion Artifact: The distortion of the signal with the subject’s movement is known as motion artifact [63,64]. Motion artifact can cause the signal baseline to vary with time at frequencies of 1–15 Hz. Figure 8A depicts this behavior. Motion artifact has many causes.
    - When the body is in motion, e.g., jogging or even during respiration, the electrode moves relative to the electrolyte. This movement can cause the electrode surface to see different electrolyte concentrations over time. Since $V_{HC}$ depends on the electrolyte concentration near the electrode’s surface, the subject’s movement results in $V_{HC}$ and thus $V_{BE}$ to vary [1,2,41,65,66].
    - When the body is in motion, the skin undergoes mechanical deformations (e.g., stretching), which causes $V_{SC}$ and thus $V_{BE}$ to vary [67,68].
    - Body movements can also cause the electrode cables to move relative to the electrode. These movements result in friction between the cable and the electrode, generating friction-induced electrical noises known as triboelectric noise that add to $V_{BE}$ [69,70].
  (b) Environmental Interference: Environmental noise sources such as AC mains near the body can potentially couple currents to the body [71]. When that happens, the currents can traverse through the electrode–body interface and create a time-varying voltage drop across $Z_{BE}$, that adds to $V_{BE}$. The currents also polarize the electrode and change its $V_{HC}$, modifying $V_{BE}$ further. Additionally, the currents flowing through the body can change the body’s potential. Since signals used in human-body sensing and communication use the body’s potential as the reference, any variation in the body’s potential will reflect in the signal measurements. Figure 8B shows the likely environmental noise sources that can interfere with the body and cause the signal baseline to wander over time.
3. Signal Attenuation and Dispersion: When signals pass through the interface, due to non-zero impedance $Z_{BE}$, a part of the signal drops across $Z_{BE}$, attenuating the output. Signal dispersion occurs due to the capacitive component of $Z_{BE}$, which results in different attenuation of low-frequency components than the high-frequency components of the signals. This frequency dependent transfer function through the body–electrode interface also depends on the termination impedance.

### 2.4. Practical Electrodes: Form Factors and Materials

**Figure 9:**
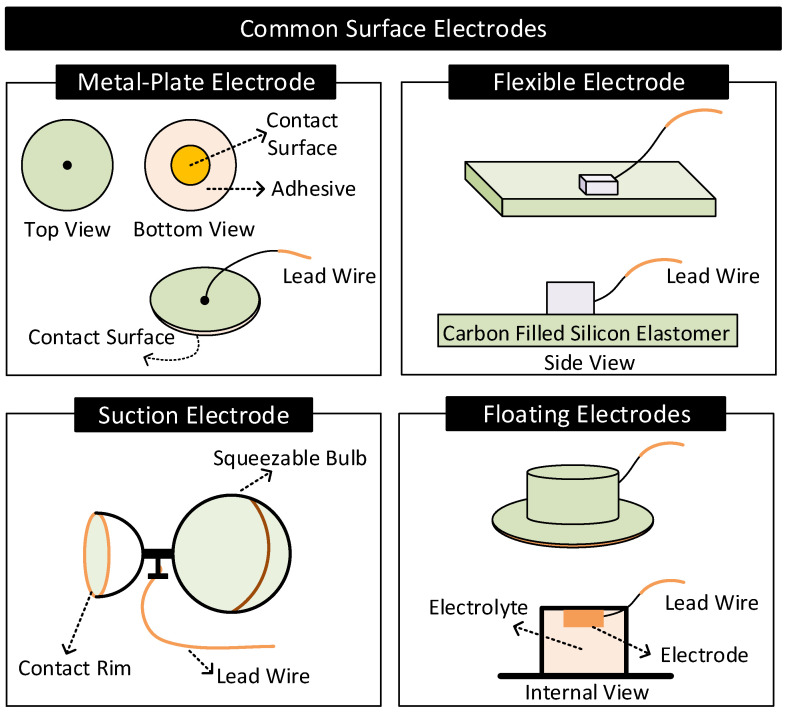
Common surface electrodes.

**Figure 10:**
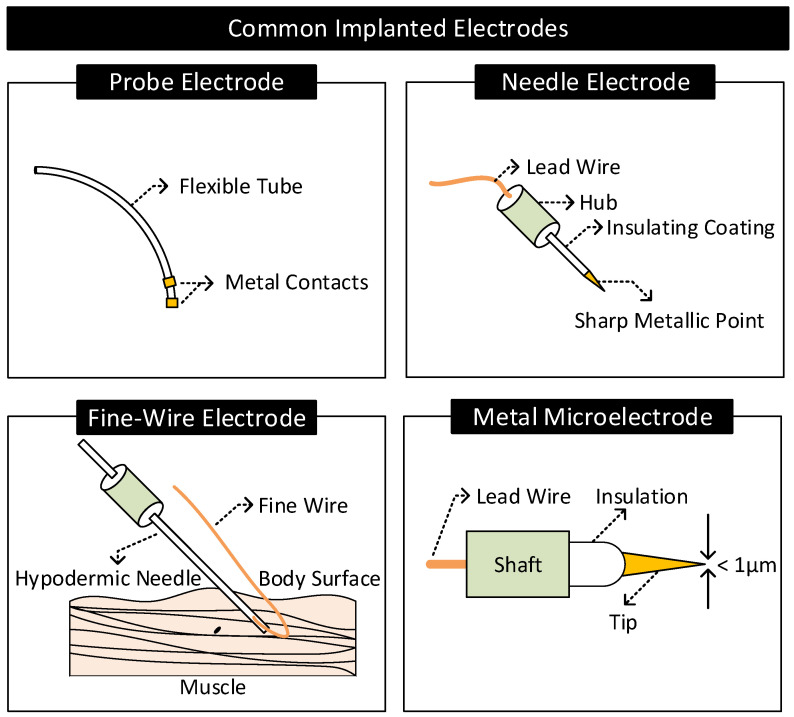
Common implanted electrodes.

Practical electrodes are available in a variety of forms and materials. Figure 9 and Figure 10 shows some common surface and implanted electrodes, respectively. They are as follows.

#### 2.4.1. Surface Electrodes

- Metal-Plate Electrodes: They consist of a metal plate with an attached lead wire [1,57]. They are fixed on the body with or without electrolytes using adhesives or surgical tapes. Metal-plate electrodes are highly prone to motion artifacts. The subject’s movements can cause electrolyte concentration near the electrode to vary, causing variations in the electrode’s half-cell potential.
- Flexible Electrodes: They consist of a thin metal foil of thickness less than a micrometer mounted on a flexible substrate such as polyester or polyimide [2]. Due to the flexibility, the electrode easily conforms to the shape of the subject’s body, improving contact impedance and enhancing the subject’s experience of wearing the electrode. Additionally, the thin metal foil allows X-rays to pass through, allowing X-ray diagnosis without removing electrodes [2].
- Suction Electrodes: They are primarily used for diagnostic purposes as they can be easily attached and transferred from one location to another on the body. They make contact with the skin surface via a suction cup. Suction electrodes are relatively large compared to metal-plate electrodes; however, the body contacts only the rim of the suction cup, resulting in high contact impedance [1,2].
- Floating Electrodes: They consist of an electrode recessed in a cup of electrolyte gel. The cup is then attached to the body using a medical-grade adhesive. Floating electrodes are less prone to motion artifacts. When the body moves, the concentration of the electrolyte near the skin may vary. However, the electrolyte near the electrode is less affected as the electrode resides at a distance away from the skin, ensuring relatively stable half-cell potential [1,2].

German silver (a nickel–silver alloy), silver, gold, platinum, and Ag/AgCl are the common materials used for making surface electrodes [2]. Among these, Ag/AgCl has low and stable half-cell potential, interface impedance, and noise and hence is preferred the most. The Ag/AgCl electrode consists of a metallic silver base coated with a layer of AgCl. The electrode contacts the body via a layer of electrolytes rich in Cl${}^{-}$ (or sweat). In this arrangement, AgCl act as a bridge connecting the metallic silver with the electrolytes [1,2,72].

#### 2.4.2. Implanted Electrodes

- Probe Electrodes: These electrodes are made of long flexible tubes with a metallic tip at the end. They are inserted in body cavities that occur naturally or through surgical procedures. The metal tip is connected to the external circuitry through a lead wire running internally through the tube [2].
- Needle Electrodes: They are rigid electrodes typically made of stainless steel with a sharp-pointed edge of diameter 100–500 μm [1,2,73]. Their design allows it to penetrate the top layers of the skin and make contact with the internal body tissues. Needle electrodes are often coated with an insulating material throughout their surface except at the tip for localized signal transfers.
- Fine-Wire Electrodes: They are flexible single-strand wires with a diameter of 25–125 μm [1,2]. Like needle electrodes, they are also coated with an insulating material throughout its surface except at the tip for localized signal transfers. Often, a hypodermic needle inserts these electrodes into the region of interest. The tip of these electrodes is bent in the form of a hook to prevent it from moving upon insertion.
- Microelectrodes: They are electrodes with a tip diameter of less than a micrometer used for transferring signals at the cellular level [74] (e.g., to sense the neural activity in the brain). They are of different types: glass micropipettes, metal microelectrodes, and solid-state microprobes [75]. Metal microelectrodes are similar in construction to a needle electrode but with their tips tapered to micrometer widths through an electrochemical etching process.

Since they are in direct contact with body tissues and bodily fluids, materials used for making implanted electrodes need to be biocompatible (e.g., non-toxic and non-inflammatory), mechanically durable, and chemically inert to avoid any adverse health conditions [76]. For instance, chemical reactions, such as corrosion or faradaic reactions between the electrode and bodily fluids in the brain, may cause unwanted stimulation of neurons leading to side effects such as brain seizures or neural damage [76,77]. For these reasons, the construction of implanted electrodes, specifically those targeting the brain region, uses chemically inert noble metals (e.g., platinum or iridium) or capacitor electrodes made by coating a layer of dielectric material on a metal surface that does not exhibit faradaic reactions [77].

## 3. Biopotential Sensing

Biopotential sensing uses electrodes in contact with the body to sense signals from within the body to infer bodily activities. The first part of this section gives a brief background on biopotential sensing. The second part of this section discusses the impact of the body–electrode interface on biopotential sensing.

### 3.1. Background

**Figure 11:**
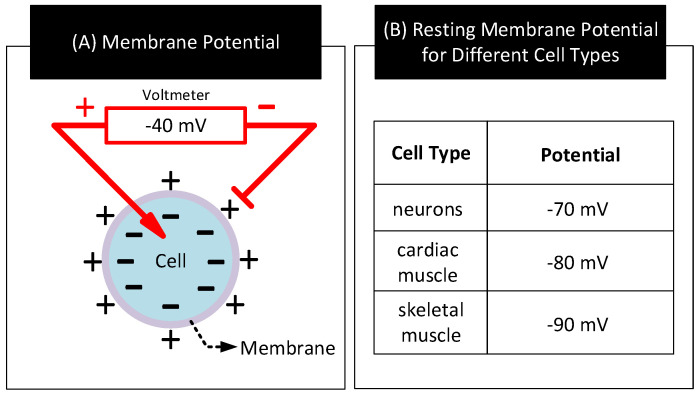
(**A**) A cell’s membrane potential. (**B**) Membrane potential values for three different cell types in their resting state [1,79].

In living cells, a difference in potential exists between the interior and exterior walls of the cell membrane. This difference in potential known as membrane potential is a result of the difference in concentration of ions (e.g., K${}^{+}$, Na${}^{+}$, Cl${}^{-}$) present in the fluids contained and surrounding the cell membrane [78]. The magnitude and polarity of the membrane potential depend on the type and state of the cell. Figure 11 shows the schematic diagram for measuring a cell’s membrane potential and typical values for the membrane potential for three different cell types in their resting state.

**Figure 12:**
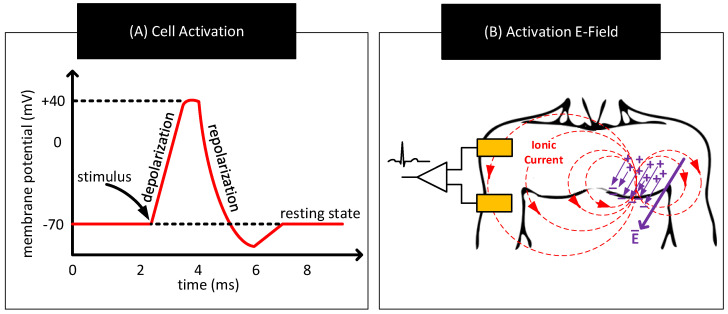
(**A**) Representative waveform showing temporal variations in the cell’s membrane potential when an external stimulus activates the cell. (**B**) Formation of an electric dipole, electric fields, and ionic currents in response to bodily activities triggering synchronous activation of a large number of cells.

Bodily activities such as heartbeat, contracting muscles, and neuron firing can alter the resting state of certain cells known as excitable cells by applying energy to their cell membrane. When that happens, ions from fluids outside the cell membrane enter the cell and depolarizes it. Depolarization of a cell occurs when the concentration of ions inside the cell changes from its resting state value and alters the cell’s resting state membrane potential towards opposite polarity. Depolarization of a cell can reverse the polarity of its membrane potential albeit for a short duration (≈few milliseconds) [1,80]. When cells detect depolarization, they close channels in the membrane to stop any further inflow of ions. Further, they open up channels in the membrane to drain out the excess ions accumulated inside the membrane. However, these actions to repolarize the cell take time, and often results in a characteristic waveform as shown in Figure 12A.

The aforementioned bodily activities often trigger depolarization and repolarization in a large number of cells. Though the ionic currents in these individual cells circulate within a small region, their combined effect can result in an electric dipole and electric field that is sufficiently strong to create measurable ionic currents near the surface of the body [81]. Biopotential sensing refers to the measurement of these ionic currents to infer bodily activities. Figure 12B shows a sample scheme to measure these ionic currents. The scheme uses a pair of electrodes on the body and continuously measures the potential difference across them to track the ionic currents.

**Figure 13:**
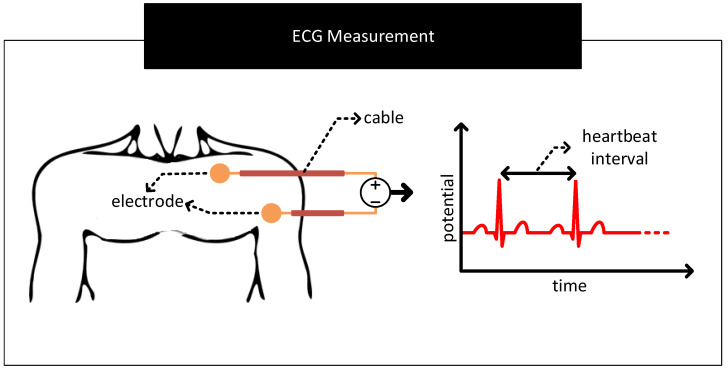
A sample scheme to measure human heart activity. The scheme uses two electrodes attached to the surface of the chest and separated by a distance to measure the biopotential signals associated with the heart activity. Differencing the signal voltages from the electrodes yields the characteristic electrocardiogram (ECG) waveform.

Figure 13 shows a simplified scheme for sensing human heart activity consisting of a pair of electrodes attached to the surface of the chest and separated by a distance. Differencing the potential across these electrodes yields the electrocardiogram (ECG), a biopotential signal representing human heart activity [82]. Physicians may extend this scheme with up to ten electrodes positioned at different locations over the body and measure potential differences between them in pairs to generate a more accurate representation of the heart activity [25]. Physicians use ECG to detect abnormal heart activities and to diagnose heart-related diseases [83,84,85,86].

### 3.2. Challenges

We discuss, in Section 2.3, the common factors that can contribute to the distortion of signals passing through the body–electrode interface. This section further describes these factors in the context of sensing biopotential signals. Throughout this section, for the descriptions, we use ECG sensing as the example.

#### 3.2.1. Contact Potential

**Figure 14:**
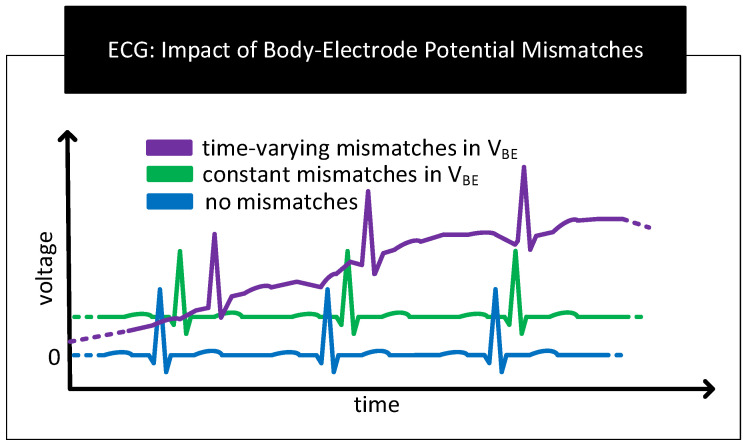
Representative ECG graphs in the absence and presence of mismatches in contact potentials. In the presence of time-varying mismatches, the baseline of the ECG graph wanders over time.

1. Restricted Amplification: The contact potential of several electrode–electrolyte interfaces used in biopotential sensing is in the order of hundreds of millivolts [2]. In contrast, biopotential signals are in the order of tens of microvolts to a couple of millivolts [87]. For the biopotential sensing circuit, the contact potential appears as a large DC offset in series to the feeble biopotential signal. Here, the large DC offset poses the following problem. It limits the amount of amplification the front-end amplifiers of the measurement circuit can apply to increase the SNR of the biopotential signal measurements. To give an example, if the contact potential is 200 mV and the biopotential signal is 1 mV, then for a 5 V amplifier that supports rail-to-rail operation, the maximum possible gain that can be set is lower than ≈5 V/200 mV = 25. Here, gain settings higher than 25 will result in the output of the front-end amplifier to stuck at rail voltage distorting or eliminating biopotential signals. Note that if the DC offset is zero in the previous case, the gain setting of the front-end amplifier could have been as high as ≈5000.
2. Baseline Wander: In the scheme to measure ECG in Figure 13, if the contact potentials at each electrode to skin contact are the same, then the resulting output which is derived from the difference block will not contain any artefacts of contact potentials. However, in practice, there exist mismatches in the contact potentials. The mismatch is often due to electrode/electrolyte manufacturing variations or due to skin chemistry being different at different parts of the body. The mismatch will now be seen along with the biopotential signal at the output of the difference block. If the mismatch in contact potentials remains the same, then it can be removed from the output by subtracting the output with a constant. However, in practice, the electrode contact potentials and their mismatches varies slowly over time, making it challenging to separate it from biopotential signals having significant low-frequency components. The aforementioned mismatches result in biopotential signal measurements to ride over a baseline that slowly wanders over time, giving the problem its name *baseline wander*. Figure 14 shows representative ECG waveforms in the presence and absence of mismatches in contact potentials.
3. Motion Artefact: The movement of the human body during respiration, while talking or while being stressed, can cause the skin near the electrodes to stretch/deform, resulting in time-varying mismatches in contact potentials [88,89]. These mismatches act as a source of noise and interference in biopotential signal measurements known as motion artefacts. Motion artefacts cause low-frequency fluctuations in the baseline of the ECG measurements which are often difficult to remove because their frequencies overlap with the frequency components of the biopotential signals.

#### 3.2.2. Contact Impedance

**Figure 15:**
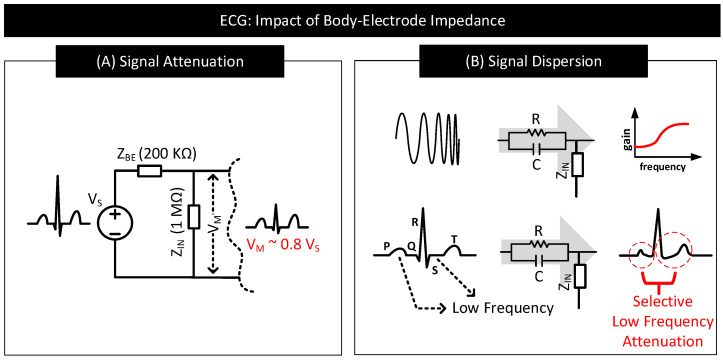
(**A**) Impact of contact impedance on biopotential signal measurement. When contact impedance is 20% of the input impedance of the measurement circuit, then only ≈80% of the signal ($V_{s}$) drops at the input ($V_{in}$) of the measurement circuit. (**B**) Distorted ECG waveform with P, S and T waves deformed and S-T segment modified due to significant attenuation in the low-frequency signal components.

1. Signal Attenuation: When measuring the biopotential signal, contact impedance acts as the source impedance of the biopotential source. If the contact impedance is significant in comparison to the input impedance of the measurement circuit, then a considerable part of the signal will be attenuated. Figure 15A depicts this behaviour.
2. Signal Dispersion: Signal attenuation per se is not a big problem if all the frequency components constituting the biopotential signal experience attenuation by the same amount. Mere amplification of the signal can reverse the effect of attenuation. However, in practice, low-frequency components of biopotential signals suffer higher degrees of attenuation due to the capacitive component of the contact impedance offering higher resistance to low-frequency signal components. The result is dispersion in biopotential signal readings that are difficult to fix. Figure 15B shows a sample contact impedance consisting of a resistor in parallel to a capacitor, its bode plot, and how it affects the low-frequency, P, S, and T regions of ECG.

#### 3.2.3. External Interferences

**Figure 16:**
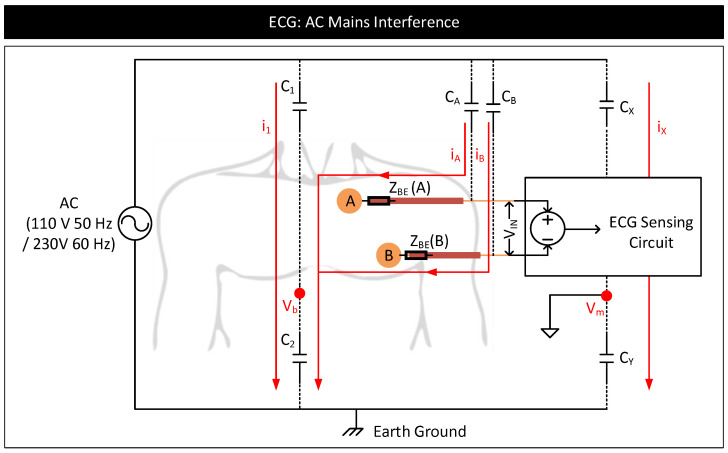
AC mains interference model of an ECG measurement system.

External interferences such as AC mains near to a biopotential signal measurement system can capacitively couple current to the human body and to the measurement circuit [90,91]. Figure 16 depicts an ECG measurement system showing the coupling of currents from AC main lines to the human body and to the ECG measurement circuit. In the figure, $i_{A}$ and $i_{B}$ are the currents capacitively coupled from the mains to the electrode cables through parasitic capacitors $C_{A}$ and $C_{B}$. These currents flow through the cable to the electrode leads and then through the body to the earth. If there exists a mismatch in these currents or contact impedances of the electrodes, then the current flows result in a differential signal that drops across the input of the measurement circuit and acts as a source of low-frequency (50/60 Hz) noise in the biopotential signal measurement. The magnitude of this noise signal, $N_{s}$, is proportional to the coupling currents, $i_{A}$, $i_{B}$, contact impedance of electrode-A ($Z_{BE}\left(A\right)$), contact impedance of electrode-B ($Z_{BE}\left(B\right)$) and their mismatches. $N_{s}$ can be calculated as follows [90,92,93].

$$N_{s}=i_{A}Z_{BE}\left(A\right)-i_{B}Z_{BE}\left(B\right)$$

External interferences can also cause common mode-induced noise in biopotential signal measurements [90]. In Figure 16, the current $i_{1}$, coupled from the AC mains to the body through the parasitic capacitor, $C_{1}$, can modulate the body potential, $V_{b}$, at 50/60 Hz with respect to the earth’s ground. Similarly, current, $i_{x}$, coupled from AC mains to the measurement system through the parasitic capacitor, $C_{x}$, can modulate the reference ground potential of the measurement system, $V_{m}$, with respect to the earth’s ground. Here, $V_{b}$ and $V_{m}$ appear common to +ve, and −ve terminals of the measurement circuit, contributing to a common-mode voltage, $V_{b}-V_{m}$. See the equations below. In the equations, $V_{+}$ and $V_{-}$ represent the voltages at the +ve and −ve terminals, respectively, of the measurement circuit with respect to $V_{m}$. $V_{A}$ and $V_{B}$ represent the biopotential signal voltages at the electrode-A and electrode-B, respectively, with respect to $V_{b}$.

$$V_{+}=\left(V_{A}+V_{b}\right)-V_{m}=V_{A}+\left(V_{b}-V_{m}\right)$$

$$V_{-}=\left(V_{B}+V_{b}\right)-V_{m}=V_{B}+\left(V_{b}-V_{m}\right)$$

In the ideal situation, since $V_{b}-V_{m}$ appear common to both the terminals of the circuit, the presence of non-zero $V_{b}$ or $V_{m}$ or their fluctuations should not affect the output of the circuit measuring the difference between $V_{+}$ and $V_{-}$. However, in reality, $V_{b}$ and $V_{m}$ and their fluctuations affect the output. Suppose the common mode rejection ratio (CMRR) of the measurement circuit is poor [94]. In that case, fluctuations in $V_{b}$ and $V_{m}$ cause variations in the common-mode voltage, resulting in common mode to differential conversions that appear as a low-frequency noise in the signal measurements [95]. Further, if $V_{b}$ and $V_{m}$ are significant, it can saturate the output of the front-end amplifiers of the measurement circuit, distorting or clipping the biopotential signals [96].

## 4. Human Body Communication

Human body communication (HBC) uses electrodes in contact with the body to transfer signals from one part of the body to the other through body tissues [3,4,5,6,7,8,9,10,11,12,13]. The first part of this section gives a brief background on HBC. The second part of this section discusses the impact of the body–electrode interface on HBC performance.

### 4.1. Background

**Figure 17:**
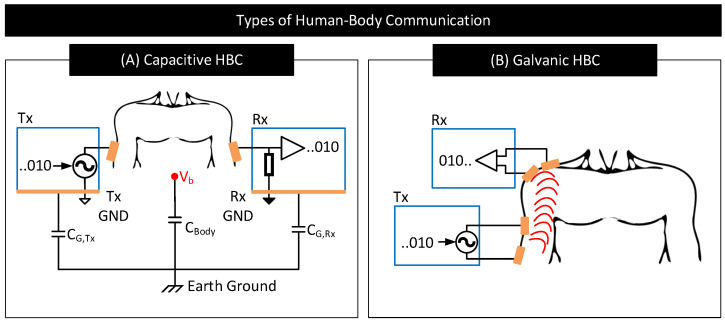
Simplified schematic of (**A**) capacitive HBC and (**B**) galvanic HBC.

HBC uses the human body as a channel to transfer data between a transmitter and a receiver. Often, HBC is used to interconnect on-body devices, in which case, both the transmitter and the receiver will reside on the body. HBC is of two types: capacitive and galvanic [3]. They differ in the methods used to couple the signal to the body and sense it from the body. Figure 17 shows a simplified schematic of capacitive and galvanic HBCs.

In capacitive HBC, the transmitter (Tx) uses a single electrode to couple signals to the body [97]. The transmitter uses this electrode to modulate the body potential ($V_{b}$) to communicate data bits. For example, the transmitter may apply a higher voltage to the electrode and raise the body potential to convey bit 1 and apply a lower voltage to the electrode and lower the body potential to convey bit 0. The receiver (Rx) uses a single electrode to sample the body potential and infer the transmitted data bits. At lower frequencies, $V_{b}$ remains more or less the same along the whole body, which enables the receiver to pick the transmitted signal with more or less the same signal quality from anywhere on the body [98]. Capacitive HBC is thus suited for applications requiring longer body transmission distances. Nevertheless, capacitive HBC is highly prone to external interferences as follows. In capacitive HBC, the SNR of the received signal depends on the strength of the parasitic capacitive coupling between the floating grounds of the transmitter and the receiver with the earth’s ground, i.e., the SNR of the received signal depends on $C_{G,Tx}$ and $C_{G,Rx}$ [98,99]. The higher their values, the higher the SNR of the received signal. However, $C_{G,Tx}$ and $C_{G,Rx}$ are usually weak and vary with the presence of nearby metallic objects, water, and the subject’s movements.

In galvanic HBC, the transmitter (Tx) uses a pair of electrodes to couple signals to the body [97]. The transmitter uses these electrodes to generate fields inside the body and modulate their strengths to communicate data. For instance, the transmitter may generate a strong field to convey bit 1 by applying a large voltage across the electrodes or generate a weak field to convey bit 0 by lowering the voltage across the electrodes. The resultant field is sampled at the receiver (Rx) through a pair of electrodes to decode the transmitted data. In galvanic HBC, often signal frequency is set low (e.g., <1 MHz [100]) to confine electric fields within the body, avoiding signal leakage to the environment and ensures that communication is less prone to external interferences in comparison to capacitive HBC. However, because the transmitting electrodes in galvanic HBC are close to each other, the generated fields often concentrate near/around these electrodes and cause the field strength to drop drastically with the distance from the transmitter. Galvanic HBC is thus not preferred for long-distance body communication.

Note: In practice, both capacitive and galvanic HBCs use high-frequency carrier signals to communicate data through the body. The HBC transmitter modulates carrier signal parameters such as frequency, amplitude, or phase to convey the information. The use of a carrier signal allows shifting HBC’s frequency of operation to avoid possible external interferences. Authors in [3] provides a comprehensive survey of different channel modulation techniques used for HBCs (e.g., OOK, FSK, QPSK, and BPSK) and the criteria used in the referenced works to select a particular channel modulation technique and carrier frequency based on channel loss measurements [5,12,101,102,103,104,105].

### 4.2. Challenges

Electrodes in HBC are used for both coupling signals to the body and sensing signals from the body. Their characteristics significantly influence the performance of HBC. In this part, we discuss the effects of electrode–body contact impedance, electrode configuration, and external interferences on capacitive and galvanic HBC performance.

#### 4.2.1. Contact Impedance

**Figure 18:**
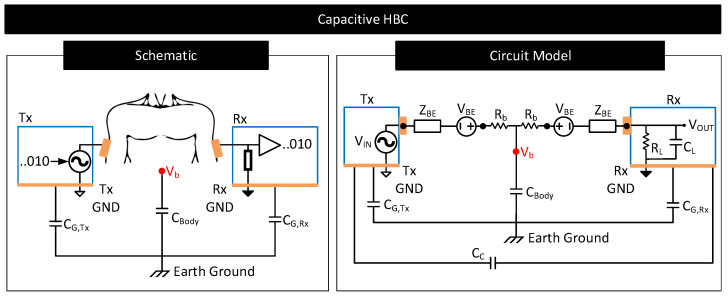
Simplified schematic and circuit model of capacitive HBC. Circuit elements $V_{BE}$ and $Z_{BE}$ model the contact potential and impedance of the body–electrode interface. Circuit element $R_{b}$ models the resistance of the conductive core of the body. Circuit element $C_{C}$ represents the inter-device coupling capacitance between the ground electrodes of Tx and Rx [107]. Parallel combination of resistance, $R_{L}$, and capacitance, $C_{L}$, model the termination impedance [106].

**Figure 19:**
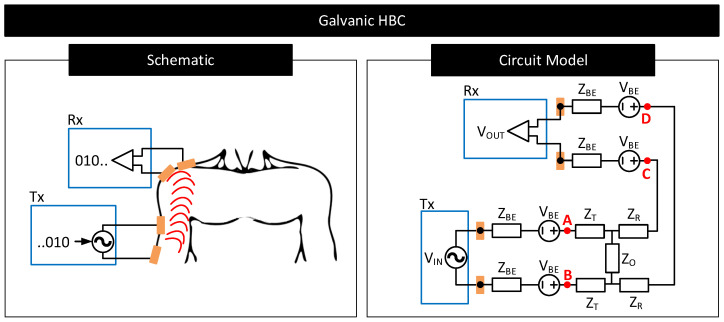
Simplified schematic and circuit model of galvanic HBC. Circuit elements $V_{BE}$ and $Z_{BE}$ model the contact potential and impedance of the body–electrode interface. Circuit elements $Z_{T}$, $Z_{O}$ and $Z_{R}$ are the Z-port representation of the impedances of the conductive core of the body [3,102].

The non-zero contact impedance of the body–electrode interface can affect the performance of capacitive and galvanic HBCs as follows:

1. Capacitive HBC: Figure 18 shows a simplified schematic and circuit model of capacitive HBC with body–electrode contact impedances [6]. At higher operating frequencies, when the contact impedances are higher in comparison to $C_{G,Tx}$ and $C_{G,Rx}$, at the transmitter side, only a part of the potential applied to the transmit electrode, $V_{IN}$, drops across the bulk of the body ($C_{Body}$) which results in a lower $V_{b}$. Further, due to higher contact impedance at the receiver side, the measured body potential at the receiver will be lesser than the actuals (i.e., $V_{OUT}$<$V_{b}$). In short, at higher operating frequencies, the interface contact impedance deteriorates the received signal strength. At lower operating frequencies (e.g., <1 MHz), the contact impedances in Figure 18 are often negligible in comparison to parasitics $C_{G,Tx}$, $C_{G,Rx}$ and $C_{Body}$ [98] and thus their impact on the received signal is minimal provided that the signal termination impedance $R_{L}\|C_{L}$ is high. However, if the termination impedance is lower or comparable to contact impedance, considerable signal attenuation can occur even at lower operating frequencies [106].
2. Galvanic HBC: Figure 19 shows the circuit model of galvanic HBC with body–electrode impedances as in [102]. Here, the fields generated in the conductive core of the body are dependent on the potential difference across A and B, i.e., $V_{AB}$. In the presence of contact impedance, only a part of the differential voltage applied across the transmitter electrode pair drops across A and B, i.e., $V_{AB}$<$V_{IN}$, which lowers the strength of the field generated in the body. Consequently, the potential generated across C and D, i.e., $V_{CD}$, and thus $V_{OUT}$, reduces. In short, the received signal strength deteriorates in the presence of contact impedances.

#### 4.2.2. Electrode Configuration

1. Capacitive HBC:
  (a) Ground Electrodes: The size, shape, and placement of the floating ground electrodes of the transmitter and receiver in capacitive HBC affect their parasitic capacitances to the earth’s ground ($C_{G,Tx}$ and $C_{G,Rx}$) and the receiver-side load capacitance ($C_{L}$) in Figure 18 [108,109]. Note that the received signal strength in capacitive HBC depends on these capacitances. Higher values of $C_{G,Tx}$ and $C_{G,Rx}$ and lower values of $C_{L}$ are desired for reducing HBC channel loss [5,98]. When the size of the ground electrodes is large or when they are close to the earth’s ground, $C_{G,Tx}$ and $C_{G,Rx}$ increase. However, increasing the size of electrodes results in bulky transmitter and receiver designs, making it difficult for the user to move freely. Further, many applications fix the site to place the transmitter and receiver; meaning, the options to place the ground electrodes close to the earth’s ground are limited. Moreover, increasing the size of the receiver-side ground electrode increases the load capacitance, $C_{L}$, which reduces the received signal strength.
    The distance between the transmitter and the receiver ground electrodes and their relative angle of orientations also matter [97,108]. At lower distances and when the ground electrodes of the transmitter and receiver are parallel, the inter-device coupling capacitor, $C_{C}$, is higher (See Figure 18). Higher $C_{C}$ results in a part of the signal returning through $C_{C}$ instead of through the relatively high impedance path $C_{G,Tx}$ and $C_{G,Rx}$ improving the channel quality [107]. Again, applications generally dictate the locations and orientations for the transmitter and receiver ground electrodes; meaning, the options to increase $C_{C}$ is limited.
  (b) Signal Electrodes: In capacitive HBC, signal electrodes are placed in direct contact with the body. The size of these electrodes dictates their contact impedance. For instance, an electrode with a large surface area increases the capacitive component of the contact impedance [6]. A higher capacitive component is preferred when operating frequencies are high, as it lowers the impedance of the forward signal path. However, having large-sized signal electrodes can make the transmitter and receiver designs bulky and increase channel loss due to higher $C_{L}$ at the receiver side.
2. Galvanic HBC:
  - Ideal Galvanic Behaviour: Here, both the pair of electrodes at the transmitter and receiver sides are signal electrodes. They are placed in direct contact with the body (see Figure 17) and exited with signals often at low frequencies to ensure that the signal forward and return paths are well defined and confined within the body (see Section 4). For this reason, and since there exist no floating ground electrodes, the performance of galvanic HBC at low frequencies is independent of the position of the electrodes to the earth’s ground, the parasitics of the electrodes to the earth’s ground, or the shape of the electrode [3,102]. However, the distance between the transmitter and receiver electrodes and their orientation matters. The received signal strength drastically drops with increasing transmitter-receiver electrode distance [102,110]. Received signal strength also drops if the transmitter-side and receiver-side electrodes are facing away from each other.
  - Non-Ideal Galvanic Behaviour: At significantly longer distances between the transmitter and the receiver, i.e., when the transmitter to the receiver distance is significant to the distance of separation of transmitter and receiver electrode pair, signal losses tend to stabilize. In this realm, the galvanic HBC channel behaves similar to capacitive HBC, with losses dependent on the electrodes’ parasitics, geometry, and configuration. Authors in [102] attribute this behavior to the presence of a non-zero common mode in the transmitted signal owing to the mismatches in the transmitter electrode parasitics and the subsequent common mode to differential-mode conversion at the receiver side.

#### 4.2.3. External Interferences

External interferences in HBC can cause undue variations in the received signal strengths, making it challenging for the receiver to decode data [111].

1. Capacitive HBC: Since the signal transfer in capacitive HBC is via modulating body potential, $V_{b}$, any external source capable of modulating $V_{b}$ acts as a source of interference. AC mains or nearby high-frequency switch-mode supplies are likely sources of interference [71] as they can modulate $V_{b}$ (see Section 3.2.3). Further, since the signal return path in capacitive HBC is through the parasitic capacitances $C_{G,Rx}$, $C_{G,Tx}$, and $C_{C}$, any source that can vary these capacitances can also act as a likely source for interference. Possible such sources include the presence of nearby wall wires, conductive objects, water, or humans [99]. The presence of grounded metallic objects in contact with the body is a strong source of interference. Such a condition drives $V_{b}$ to earth’s potential. When that happens, the transmitter electrodes cannot modulate $V_{b}$ and thus cannot transmit signals.
2. Galvanic HBC: As mentioned in Section 4.1, at low frequencies, signal leakage to the environment is minimal in galvanic HBC. For this reason, galvanic HBC at low frequencies is less prone to external interferences that affect electrode parasitics. However, external interferences such as AC mains or nearby SMPS can vary the potential of the body and the local ground potential of the receiver circuit similarly to Figure 16. These potential variations may appear as common-mode noise to the receiver-side measurement circuit and lower the SNR of the measured signal.

## 5. Conclusions

We review in this work the physics of the body–electrode interface, how it distorts the signals passing through the interface, and how these distortions affect biopotential sensing and human body communication (HBC). We find that the body–electrode interface characteristics, such as its impedances and potential, rely on many factors. The electrode properties, the electrode–body configuration, and skin properties can affect the body–electrode interface. Although an ideal body–electrode interface is expected to transfer current from body to electrode and vice versa without hindrance, practical body–electrode interfaces due to definite impedances and potentials often hinder current flow, which results in distortions of signals passing through the interface. Several factors contribute to signal distortion. The presence of contact potential and its variation, frequency-dependent contact impedance, relative movement of the body to the electrode, the polarization of electrodes due to the current flowing through the contact impedance, and external interferences from AC mains can all contribute to signal distortion. We find that these signal distortions across the body–electrode interface can be troublesome in sensing and communication applications. Specific to biopotential sensing applications, the interface potential can limit the gain of the front-end amplifier, restricting the SNR of the received signal. Its time-dependent variation can cause the baseline of the biopotential signals to vary over time. The interface impedance can cause attenuation and dispersion of biopotential signals. The interface’s impedance can also attract external interferences and add unwanted noise to the biopotential signals. Specific to HBC, the interface impedance can cause attenuation of the transmitted signals, limiting the received signal strength and thus restricting the bitrate of communication. Electrode configuration, i.e., the placement of the electrode on the body, its parasitics to the earth’s ground, and nearby objects, can also affect communication signal quality. External interferences also affect HBC. We find that capacitive HBC is more dependent on external interference than galvanic HBC due to the former’s dependency on electrode parasitics.

## Figures and Tables

**Table 1:** Common biopotential signals, their sources and applications.

Biopotential Signal	Biopotential Source	Application
Electrocardiogram (ECG) [25]	Heart activity. Measured from the body’s surface.	Diagnosis of heart related diseases.
Electromyogram (EMG) [26]	Muscular activity. Measured from the body’s surface.	Analyzis of biomechanics of body movements.
Electroencephalogram (EEG) [27]	Brain activity. Measured from the surface of the scalp.	Diagnosis of abnormal brain activities resulting from epilepsy, strokes, sleep disorders etc.
Local Field Potentials (LFP) [28]	Brain activity. Measured from brain tissues.	Responsive stimulation of brain.
Neural Spikes [24]	Neural action potential. Measured from a single neuron.	Analyzis of brain activity.

**Table 2:** Half-cell potential ($V_{HC}$) of common electrodes under standard measurement conditions [1].

Electrode	$V_{\mathrm{HC}}$ (V)	Electrode	$V_{\mathrm{HC}}$ (V)
Aluminium	−1.71	Silver Chloride	+0.23
Iron	−0.41	Copper	+0.34
Nickel	−0.23	Silver	+0.80
Lead	−0.13	Gold	+1.68

## References

[1] Button V.L.D.S.N. (2015). Electrodes for Biopotential Recording and Tissue Stimulation. Principles of Measurement and Transduction of Biomedical Variables.

[2] Neuman M.R. (1998). Biopotential electrodes. Med. Instrum. Appl. Des..

[3] Zhao J.F., Chen X.M., Liang B.D., Chen Q.X. (2017). A review on human body communication: Signal propagation model, communication performance, and experimental Issues. Wirel. Commun. Mob. Comput..

[4] Naranjo-Hernández D., Callejón-Leblic A., Lučev Vasić Ž., Seyedi M., Gao Y.M. (2018). Past results, present trends, and future challenges in intrabody communication. Wirel. Commun. Mob. Comput..

[5] Maity S., Das D., Chatterjee B., Sen S. Characterization and classification of human body channel as a function of excitation and termination modalities. Proceedings of the 2018 40th Annual International Conference of the IEEE Engineering in Medicine and Biology Society (EMBC).

[6] Maity S., He M., Nath M., Das D., Chatterjee B., Sen S. (2018). Bio-physical modeling, characterization, and optimization of electro-quasistatic human body communication. IEEE Trans. Biomed. Eng..

[7] Maity S., Chatterjee B., Chang G., Sen S. (2019). BodyWire: A 6.3-pJ/b 30-Mb/s- 30-dB SIR-tolerant broadband interference-robust human body communication transceiver using time domain interference rejection. IEEE J. Solid State Circuits.

[8] Maity S., Das D., Sen S. Wearable health monitoring using capacitive voltage-mode human body communication. Proceedings of the 2017 39th Annual International Conference of the IEEE Engineering in Medicine and Biology Society (EMBC).

[9] Lucev Ž., Krois I., Cifrek M. (2012). A capacitive intrabody communication channel from 100 kHz to 100 MHz. IEEE Trans. Instrum. Meas..

[10] Park J., Garudadri H., Mercier P.P. (2016). Channel modeling of miniaturized battery-powered capacitive human body communication systems. IEEE Trans. Biomed. Eng..

[11] Cho N., Yoo J., Song S.J., Lee J., Jeon S., Yoo H.J. (2007). The human body characteristics as a signal transmission medium for intrabody communication. IEEE Trans. Microw. Theory Tech..

[12] Song Y., Hao Q., Zhang K. (2013). Review of the modeling, simulation and implement of intra-body communication. Def. Technol..

[13] Callejon M.A., Naranjo-Hernandez D., Reina-Tosina J., Roa L.M. (2013). A comprehensive study into intrabody communication measurements. IEEE Trans. Instrum. Meas..

[14] Das D., Maity S., Chatterjee B., Sen S. (2019). Enabling covert body area network using electro-quasistatic human body communication. Sci. Rep..

[15] Fahier N., Fang W.C. An HBC-based continuous bio-potential system monitoring using 30MHz OOK modulation. Proceedings of the 2017 IEEE Biomedical Circuits and Systems Conference (BioCAS).

[16] Zio Monitoring. https://www.irhythmtech.com/providers/zio-service/zio-monitors.

[17] Home—VitalConnect. https://vitalconnect.com/.

[18] Kardia Mobile EKG|Omron Healthcare. https://omronhealthcare.com/products/kardia-mobile-ekg-ac009uac/.

[19] Portable EKG Monitor—Instant EKG Analysis with APP—Wellue Health. https://getwellue.com/products/duoek-hand-held-wearable-ekg-tracker.

[20] BardyDx. https://www.bardydx.com/.

[21] MAX-ECGMONITOR Wearable ECG and Heart Monitor Evaluation and Development Platform—Maxim Integrated. https://www.maximintegrated.com/en/products/interface/sensor-interface/MAX-ECGMONITOR.html.

[22] Maity S., Modak N., Yang D., Avlani S., Nath M., Danial J., Das D., Mehrotra P., Sen S. A 415 nw physically and mathematically secure electro-quasistatic hbc node in 65nm cmos for authentication and medical applications. Proceedings of the 2020 IEEE Custom Integrated Circuits Conference (CICC).

[23] Chen C.H., Mak P.I., Zhang T.T., Vai M.I., Mak P.U., Pun S.H., Wan F., Martins R. A 2.4 Hz-to-10 kHz-tunable biopotential filter using a novel capacitor multiplier. Proceedings of the 2009 Asia Pacific Conference on Postgraduate Research in Microelectronics & Electronics (PrimeAsia).

[24] Harrison R.R. A versatile integrated circuit for the acquisition of biopotentials. Proceedings of the 2007 IEEE Custom Integrated Circuits Conference.

[25] Anderson J., DiCarlo S.E. (2000). “Virtual” experiment for understanding the electrocardiogram and the mean electrical axis. Adv. Physiol. Educ..

[26] Milner-Brown H., Stein R. (1975). The relation between the surface electromyogram and muscular force. J. Physiol..

[27] Subha D.P., Joseph P.K., Acharya R., Lim C.M. (2010). EEG signal analysis: A survey. J. Med. Syst..

[28] Maling N., McIntyre C. (2016). Local Field Potential Analysis for Closed-Loop Neuromodulation. Closed Loop Neuroscience.

[29] Biesheuvel P., Porada S., Dykstra J. (2018). The difference between Faradaic and non-Faradaic electrode processes. arXiv.

[30] Ratner B.D., Hoffman A.S., Schoen F.J., Lemons J.E. (2004). Biomaterials Science: An Introduction to Materials in Medicine.

[31] Beckmann L., Neuhaus C., Medrano G., Jungbecker N., Walter M., Gries T., Leonhardt S. (2010). Characterization of textile electrodes and conductors using standardized measurement setups. Physiol. Meas..

[32] Xiong F., Chen D., Chen Z., Jin C., Dai S. (2019). Impedance characteristics of the skin-electrode interface of dry textile electrodes for wearable electrocardiogram. Advances in Body Area Networks I.

[33] Merrill D.R., Bikson M., Jefferys J.G. (2005). Electrical stimulation of excitable tissue: Design of efficacious and safe protocols. J. Neurosci. Methods.

[34] Heikenfeld J., Jajack A., Rogers J., Gutruf P., Tian L., Pan T., Li R., Khine M., Kim J., Wang J. (2018). Wearable sensors: Modalities, challenges, and prospects. Lab Chip.

[35] Lu F., Wang C., Zhao R., Du L., Fang Z., Guo X., Zhao Z. (2018). Review of stratum corneum impedance measurement in non-invasive penetration application. Biosensors.

[36] Foulds I., Barker A. (1983). Human skin battery potentials and their possible role in wound healing. Br. J. Dermatol..

[37] Huang X., Cheng H., Chen K., Zhang Y., Zhang Y., Liu Y., Zhu C., Ouyang S.C., Kong G.W., Yu C. (2013). Epidermal impedance sensing sheets for precision hydration assessment and spatial mapping. IEEE Trans. Biomed. Eng..

[38] Clar E., Her C., Sturelle C. (1975). Skin impedance and moisturization. J. Soc. Cosmet. Chem..

[39] Yokus M.A., Jur J.S. (2015). Fabric-based wearable dry electrodes for body surface biopotential recording. IEEE Trans. Biomed. Eng..

[40] Myers A.C., Huang H., Zhu Y. (2015). Wearable silver nanowire dry electrodes for electrophysiological sensing. RSC Adv..

[41] Cömert A., Honkala M., Hyttinen J. (2013). Effect of pressure and padding on motion artifact of textile electrodes. Biomed. Eng. Online.

[42] Meng Y., Li Z., Chen J. (2016). A flexible dry electrode based on APTES-anchored PDMS substrate for portable ECG acquisition system. Microsyst. Technol..

[43] Baek J.Y., An J.H., Choi J.M., Park K.S., Lee S.H. (2008). Flexible polymeric dry electrodes for the long-term monitoring of ECG. Sens. Actuators A Phys..

[44] Yao S., Zhu Y. (2016). Nanomaterial-enabled dry electrodes for electrophysiological sensing: A review. JOM.

[45] Jeong J., Kim M., Cheng H., Yeo W., Huang X., Liu Y., Zhang Y., Huang Y., Rogers J. (2014). Capacitive epidermal electronics for electrically safe, long-term electrophysiological measurements. Adv. Healthc. Mater..

[46] Wang F., Li G., Chen J., Duan Y., Zhang D. (2016). Novel semi-dry electrodes for brain–computer interface applications. J. Neural Eng..

[47] Li G.L., Wu J.T., Xia Y.H., He Q.G., Jin H.G. (2020). Review of semi-dry electrodes for EEG recording. J. Neural Eng..

[48] Li G., Wang S., Li M., Duan Y.Y. (2021). Towards real-life EEG applications: Novel superporous hydrogel-based semi-dry EEG electrodes enabling automatically ‘charge–discharge’ electrolyte. J. Neural Eng..

[49] Li G., Zhang D., Wang S., Duan Y.Y. (2016). Novel passive ceramic based semi-dry electrodes for recording electroencephalography signals from the hairy scalp. Sens. Actuators B Chem..

[50] Li G., Wang S., Duan Y.Y. (2018). Towards conductive-gel-free electrodes: Understanding the wet electrode, semi-dry electrode and dry electrode-skin interface impedance using electrochemical impedance spectroscopy fitting. Sens. Actuators B Chem..

[51] The Wet-EEG Cap: Semi-Dry, Saline & Gel EEG Caps|Bitbrain. https://www.bitbrain.com/blog/wet-eeg-cap.

[52] Ha S., Kim C., Chi Y.M., Akinin A., Maier C., Ueno A., Cauwenberghs G. (2014). Integrated circuits and electrode interfaces for noninvasive physiological monitoring. IEEE Trans. Biomed. Eng..

[53] Rai P., Oh S., Shyamkumar P., Ramasamy M., Harbaugh R.E., Varadan V.K. (2013). Nano-bio-textile sensors with mobile wireless platform for wearable health monitoring of neurological and cardiovascular disorders. J. Electrochem. Soc..

[54] Assambo C., Baba A., Dozio R., Burke M. Determination of the parameters of the skin-electrode impedance model for ECG measurement. Proceedings of the 6th WSEAS International Conference on Electronics, Hardware, Wireless and Optical Communications.

[55] Kusche R., Kaufmann S., Ryschka M. (2018). Dry electrodes for bioimpedance measurements—Design, characterization and comparison. Biomed. Phys. Eng. Express.

[56] Liao L.D., Wang I.J., Chen S.F., Chang J.Y., Lin C.T. (2011). Design, fabrication and experimental validation of a novel dry-contact sensor for measuring electroencephalography signals without skin preparation. Sensors.

[57] Chi Y.M., Jung T.P., Cauwenberghs G. (2010). Dry-contact and noncontact biopotential electrodes: Methodological review. IEEE Rev. Biomed. Eng..

[58] Xu J., Mohan R., Van Helleputte N., Mitra S. (2018). Design and optimization of ICs for wearable EEG sensors. CMOS Circuits for Biological Sensing and Processing.

[59] Li G., Wang S., Duan Y.Y. (2017). Towards gel-free electrodes: A systematic study of electrode-skin impedance. Sens. Actuators B Chem..

[60] McAdams E., Jossinet J., Lackermeier A., Risacher F. (1996). Factors affecting electrode-gel-skin interface impedance in electrical impedance tomography. Med. Biol. Eng. Comput..

[61] Oster C.D. Proper Skin Prep Helps Ensure ECG Trace Quality. https://multimedia.3m.com/mws/media/358372O/proper-skin-prep-ecg-trace-quality-white-paper.pdf.

[62] Chen W.C., Guido K., Kiourti A. (2019). Passive Impedance Matching for Implanted Brain–Electrode Interfaces. IEEE J. Electromagn. Microw. Med. Biol..

[63] Webster J.G. (1984). Reducing motion artifacts and interference in biopotential recording. IEEE Trans. Biomed. Eng..

[64] Tankisi H., Burke D., Cui L., de Carvalho M., Kuwabara S., Nandedkar S.D., Rutkove S., Stålberg E., van Putten M.J., Fuglsang-Frederiksen A. (2020). Standards of instrumentation of EMG. Clin. Neurophysiol..

[65] Seok D., Lee S., Kim M., Cho J., Kim C. (2021). Motion artifact removal techniques for wearable EEG and PPG sensor systems. Front. Electron..

[66] Buxi D., Kim S., Van Helleputte N., Altini M., Wijsman J., Yazicioglu R.F., Penders J., Van Hoof C. (2012). Correlation between electrode-tissue impedance and motion artifact in biopotential recordings. IEEE Sens. J..

[67] de Talhouet H., Webster J.G. (1996). The origin of skin-stretch-caused motion artifacts under electrodes. Physiol. Meas..

[68] Burbank D.P., Webster J.G. (1978). Reducing skin potential motion artefact by skin abrasion. Med. Biol. Eng. Comput..

[69] Simakov A., Webster J. (2010). Motion artifact from electrodes and cables. IJECE.

[70] Triboelectric Noise in Medical Cables and Wires|Experience Molex. https://experience.molex.com/triboelectric-noise-in-medical-cables-and-wires/.

[71] Yang D., Mehrotra P., Weigand S., Sen S. (2021). In-The-Wild Interference Characterization and Modelling for Electro-Quasistatic-HBC with Miniaturized Wearables. IEEE Trans. Biomed. Eng..

[72] Li G., Wu J., Xia Y., Wu Y., Tian Y., Liu J., Chen D., He Q. (2020). Towards emerging EEG applications: A novel printable flexible Ag/AgCl dry electrode array for robust recording of EEG signals at forehead sites. J. Neural Eng..

[73] Dillingham T., Andary M., Dumitru D. (2021). Electrodiagnostic medicine. Braddom’s Physical Medicine and Rehabilitation.

[74] Zeuthen T., Worsfold P., Townshend A., Poole C. (2005). Microelectrodes. Encyclopedia of Analytical Science.

[75] Mendelson Y., Enderle J.D., Bronzino J.D. (2012). Chapter 10—Biomedical Sensors. Introduction to Biomedical Engineering.

[76] Bain L. (1986). Materials for Implantable Electrodes. MRS Bull..

[77] McCreery D., Agnew W., Yuen T., Bullara L. (1988). Comparison of neural damage induced by electrical stimulation with faradaic and capacitor electrodes. Ann. Biomed. Eng..

[78] Wright S.H. (2004). Generation of resting membrane potential. Adv. Physiol. Educ..

[79] Hopkins P.M. (2006). Skeletal muscle physiology. Contin. Educ. Anaesth. Crit. Care Pain.

[80] PASCO CI-6539A EKG SENSOR User Manual. https://manualmachine.com/pasco/ci6539aekgsensor/1700487-user-manual/.

[81] Lopez-Gordo M.A., Sanchez-Morillo D., Valle F.P. (2014). Dry EEG electrodes. Sensors.

[82] Bansal A., Joshi R. (2018). Portable out-of-hospital electrocardiography: A review of current technologies. J. Arrhythm..

[83] Ritter M.A., Kochhäuser S., Duning T., Reinke F., Pott C., Dechering D.G., Eckardt L., Ringelstein E.B. (2013). Occult atrial fibrillation in cryptogenic stroke: Detection by 7-day electrocardiogram versus implantable cardiac monitors. Stroke.

[84] Wyngaarden J.B., Smith L.H. (1982). Cecil Textbook of Medicine.

[85] Gupta M., Kurmi M.P., Sharma B.R., Chen L., Shahi R., Jian S. (2015). Clinical significance of ST segment depression in lead aVR to predict culprit artery in an acute inferior wall myocardial infarction. Nepal. Heart J..

[86] Coppola G., Carità P., Corrado E., Borrelli A., Rotolo A., Guglielmo M., Nugara C., Ajello L., Santomauro M., Novo S. (2013). ST segment elevations: Always a marker of acute myocardial infarction?. Indian Heart J..

[87] Jiang Y., Samuel O.W., Liu X., Wang X., Idowu P.O., Li P., Chen F., Zhu M., Geng Y., Wu F. (2018). Effective biopotential signal acquisition: Comparison of different shielded drive technologies. Appl. Sci..

[88] Physio Control Inc. (2015). Minimizing ECG Artifact. Physio-Control.

[89] An X., Stylios G.K. (2020). Comparison of motion artefact reduction methods and the implementation of adaptive motion artefact reduction in wearable electrocardiogram monitoring. Sensors.

[90] Babusiak B., Borik S., Smondrk M. (2020). Two-Electrode ECG for Ambulatory Monitoring with Minimal Hardware Complexity. Sensors.

[91] Acharya V. (2011). Improving Common-Mode Rejection Using the Right-Leg Drive Amplifier.

[92] Yamamoto Y. Impedance balancing analysis for power-line interference elimination in ECG signal. Proceedings of the IMTC/98 Conference Proceedings, IEEE Instrumentation and Measurement Technology Conference, Where Instrumentation is Going (Cat. No. 98CH36222).

[93] Becchetti C., Neri A. (2013). Medical Instrument Design and Development: From Requirements to Market Placements.

[94] Nash E., Devices A. (1998). Common Mode and Instrumentation Amplifiers.

[95] Dobrev D., Daskalov I. (2002). Two-electrode biopotential amplifier with current-driven inputs. Med. Biol. Eng. Comput..

[96] Koo N., Cho S. (2020). A 24.8-μW Biopotential Amplifier Tolerant to 15-VPP Common-Mode Interference for Two-Electrode ECG Recording in 180-nm CMOS. IEEE J. Solid-State Circuits.

[97] Pereira M.D., Alvarez-Botero G.A., de Sousa F.R. (2015). Characterization and modeling of the capacitive HBC channel. IEEE Trans. Instrum. Meas..

[98] Nath M., Maity S., Sen S. (2019). Toward understanding the return path capacitance in capacitive human body communication. IEEE Trans. Circuits Syst. II Express Briefs.

[99] Nath M., Maity S., Avlani S., Weigand S., Sen S. (2021). Inter-body coupling in electro-quasistatic human body communication: Theory and analysis of security and interference properties. Sci. Rep..

[100] Wegmüller M.S. (2007). Intra-Body Communication for Biomedical Sensor Networks. Ph.D. Thesis.

[101] Avlani S., Nath M., Maity S., Sen S. A 100KHz-1GHz termination-dependent human body communication channel measurement using miniaturized wearable devices. Proceedings of the 2020 Design, Automation & Test in Europe Conference & Exhibition (DATE).

[102] Modak N., Nath M., Chatterjee B., Maity S., Sen S. (2020). Bio-Physical Modeling of Galvanic Human Body Communication in Electro-Quasistatic Regime. bioRxiv.

[103] Wegmueller M.S., Oberle M., Felber N., Kuster N., Fichtner W. (2009). Signal transmission by galvanic coupling through the human body. IEEE Trans. Instrum. Meas..

[104] Seyed Mazloum N. (2008). Body-Coupled Communications-Experimental Characterization, Channel Modeling and Physical Layer Design. Master’s Thesis.

[105] Bae J., Cho H., Song K., Lee H., Yoo H.J. (2012). The signal transmission mechanism on the surface of human body for body channel communication. IEEE Trans. Microw. Theory Tech..

[106] Sen S. (2021). Wearable Health Monitoring System and Method Using Human Body Communication.

[107] Datta A., Nath M., Yang D., Sen S. (2021). Advanced biophysical model to capture channel variability for eqs capacitive hbc. IEEE Trans. Biomed. Eng..

[108] Mao J., Yang H., Zhao B. (2017). An investigation on ground electrodes of capacitive coupling human body communication. IEEE Trans. Biomed. Circuits Syst..

[109] Hnyk P., Kvarda L., Vojtech L., Neruda M., Zitta T. Electrode Shapes and Frequency Band Analysis for Human Body Communication. Proceedings of the 2018 18th International Conference on Mechatronics-Mechatronika (ME).

[110] Gao Y.M., Wu Z.M., Pun S.H., Mak P.U., Vai M.I., Du M. (2016). A novel field-circuit FEM modeling and channel gain estimation for galvanic coupling real IBC measurements. Sensors.

[111] Hwang J.H., Kang T.W., Kwon J.H., Park S.O. (2016). Effect of electromagnetic interference on human body communication. IEEE Trans. Electromagn. Compat..

